# Virus-Like Particles Are a Superior Platform for Presenting M2e Epitopes to Prime Humoral and Cellular Immunity against Influenza Virus

**DOI:** 10.3390/vaccines6040066

**Published:** 2018-09-20

**Authors:** Ki-Hye Kim, Young-Man Kwon, Young-Tae Lee, Min-Chul Kim, Hye Suk Hwang, Eun-Ju Ko, Youri Lee, Hyo-Jick Choi, Sang-Moo Kang

**Affiliations:** 1 Institute for Biomedical Sciences, Georgia State University, Atlanta, GA 30303, USA; kihyekim4282@gmail.com (K.-H.K.); ymankwon@gmail.com (Y.-M.K.); leechard75@gmail.com (Y.-T.L.); mckim001@gmail.com (M.-C.K.); hshwang33@gmail.com (H.S.H.); ej.ko226@gmail.com (E.-J.K.); youl6248@gmail.com (Y.L.); 2 Komipharm Co., Ltd., Siheung, Gyeonggi-do 15094, Korea; 3 Department of Microbiology, Chonnam National University Medical School, Hwasun-gun, Jeonnam 58128, Korea; 4 National Cancer Institute, National Institutes of Health, Bethesda, MD 20850, USA; 5 Department of Chemical and Materials Engineering, University of Alberta, Edmonton, AB T6G 2V4, Canada; hyojick@ualberta.ca

**Keywords:** priming effect, 5xM2e epitopes, virus-like particles (VLP), cross-protection, innate/adaptive immunity

## Abstract

Influenza virus M2 protein has a highly conserved ectodomain (M2e) as a cross-protective antigenic target. We investigated the antigenic and immunogenic properties of tandem repeat M2e (5xM2e) proteins and virus-like particles (5xM2e VLP) to better understand how VLP and protein platform vaccines induce innate and protective adaptive immune responses. Despite the high antigenic properties of 5xM2e proteins, the 5xM2e VLP was superior to 5xM2e proteins in inducing IgG2a isotype antibodies, T cell responses, plasma cells and germinal center B cells as well as in conferring cross protection. Mice primed with 5xM2e VLP were found to be highly responsive to 5xM2e protein boost, overcoming the low immunogenicity and protective efficacy of 5xM2e proteins. Immunogenic differences between VLPs and proteins in priming immune responses might be due to an intrinsic ability of 5xM2e VLP to stimulate dendritic cells secreting T helper type 1 (Th1) cytokines. We also found that 5xM2e VLP was effective in inducing inflammatory cytokines and chemokines, and in recruiting macrophages, monocytes, neutrophils, and CD11b^+^ dendritic cells at the injection site. Therefore, this study provides evidence that 5xM2e VLP is an effective vaccine platform, inducing cross-protection by stimulating innate and adaptive immune responses.

## 1. Introduction

Current influenza vaccines relying on immunity to the highly variable hemagglutinin (HA) antigen are not effective in providing protection against antigenic variants and potential pandemic strains. In contrast, the influenza A virus M2 ion channel protein has a highly conserved extracellular domain of 23 residues (M2e) proximal to the membrane surface [1]. Anti-M2 antibodies induced by natural infection have been reported, but the frequency and levels of M2 antibodies are low and also the data are rare for humans [2,3]. M2 itself is a very poor immunogen, probably due to the small extracellular domain of M2e, membrane proximity, and low levels of M2 on virions, as well as the major antigens HA and neuraminidase (NA) directly masking M2 from the immune system. The routes of vaccine delivery are also important. It was reported that the route of vaccine delivery impacts on the protection induced by influenza A virus conserved antigens (M2e, NP, HA2) and on vaccine adverse effects, preferring the intramuscular route over the intradermal route in a pig model [4]. Recombinant nucleoprotein-M2e vaccines were more effective in conferring protection when mice were immunized intranasally compared to the subcutaneous route [5].

Various approaches were applied to fuse multiple M2e peptides to carrier proteins or vehicles. Some representative carrier molecules or systems include hepatitis B virus core (HBVc) particles [6,7], bacterial outer membrane protein complexes [8,9], human papillomavirus L proteins [10], keyhole limpet hemocyanin [11], liposomes [12], cholera toxin subunit [13], phage Qβ-derived protein cores [14], and flagellin [15]. Multiple immunizations of mice with M2e protein conjugate vaccines were required to induce M2e antibodies despite using immunogenic carriers. The adjuvants used for M2e vaccines include complete or incomplete Freund’s adjuvant [16], cholera toxin subunits [17], monophosphoryl lipid A [13], Ribi-adjuvant, alhydrogel, or heat-labile enterotoxin [6,18,19]. These previous studies demonstrated the benefits of protection against lethal infection in mice with M2e conjugate vaccines in the presence of strong adjuvants.

Several research groups have reported the concept of recombinant influenza vaccines based on virus-like particles (VLPs) that were assembled by matrix proteins and decorated with surface influenza proteins [20,21,22]. In our previous study, we reported the construction of VLP vaccines containing a tandem repeat of M2e sequences (5xM2e) derived from human, swine, and avian origin influenza A viruses (5xM2e VLP), which could confer cross protection against H1, H3, and H5 subtype influenza viruses in the absence of adjuvants [23]. Whereas, 5xM2e soluble proteins with high M2e epitopes were not immunogenic and required adjuvants to induce M2e antibodies in mice [24]. 

In this study, we carried out in vivo and in vitro approaches to better understand possible mechanisms regarding how VLP and protein platform vaccines induce protective innate and adaptive immune responses. We found that 5xM2e VLP primed immune responses that were effectively boosted after subsequent vaccination with low immunogenic 5xM2e soluble proteins. The 5xM2e VLP was found to be highly immunogenic despite low antigenicity compared to 5xM2e proteins, prime M2e T helper type 1 (Th1) IgG2a antibodies, B cells, and Th1 type T cell responses, leading to enhanced protection. We also studied the differences between 5xM2e VLP and 5xM2e proteins in stimulating innate immune responses. 

## 2. Materials and Methods

### 2.1. Influenza 5xM2e Vaccines and Virus Reagents

**Figure A1:**
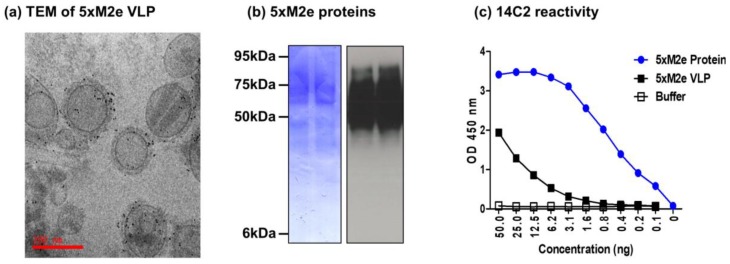
Characterization of VLP and protein of 5xM2e. (**a**) Transmission electron microscopy (TEM) of 5xM2e VLP. Bar indicates a size of 200 nm. (**b**) SDS-PAGE and Western blot of 5xM2e proteins. Oligomeric forms 5xM2e proteins were observed. (**c**) The reactivity of VLP and protein of 5xM2e to 14C2 mAb were determined by ELISA. The concentrations are indicated as coated onto the ELISA plates. Buffer: negative control.

As shown in Table 1, the 5xM2e VLP (virus-like particle) vaccine contains heterologous tandem repeats (Table 1) of two human M2e (SLLTEVETPIRNEWGSRSN), swine M2e (SLLTEVETPTRSEWESRSS), type I avian M2e (SLLTEVETPTRNEWESRSS), and type II avian M2e (SLLTEVETLTRNGWGCRCS), as previously described [23]. The 5xM2e and an oligomer-stabilizing domain GCN4 fusion protein was linked to the transmembrane (TM) cytoplasmic domain of hemagglutinin (HA) to enhance the incorporation of M2e tandem repeats into VLP [23]. Briefly, to obtain VLPs, Sf9 cells were coinfected with recombinant baculoviruses expressing influenza virus M1 and 5xM2e protein, and cultured in SF900-II serum-free medium at 27 °C. Culture supernatants were collected by centrifugation at 3000 rpm for 20 min to remove cells. The collected supernatants containing influenza VLP were further spun by ultracentrifugation at 30,000 rpm for one hour (h). The 5xM2e VLP pellets were resuspended in phosphate-buffered saline (PBS) and VLP vaccines were further purified by ultracentrifugation with sucrose gradient (20%, 30%, 60%) at 30,000 rpm for 1 h at 4 °C. The 5xM2e VLP was adsorbed onto formvar/carbon-coated copper grids (Electron Microscopy Sciences, Fort Washington, PA, USA) for 15 min. The transmission electron microscopy (TEM) picture of the VLPs consisting of M1 and 5xM2e shows spherical particles in a range of 100–180 nm (Appendix Figure A1a). In an assay of endotoxin unit (EU) tests, the VLP displayed a very low level of <1.2 EU per 10 μg VLP, which is far below the recommended level for recombinant subunit vaccines of 20 EU/mL [25].

Vaccine production in yeast cells has some merits when compared with insect or mammalian cells in terms of scaleup vaccine manufacturing and short production time, low operating costs, simple culture conditions and no mammalian or endotoxin contamination. To generate recombinant 5xM2e protein vaccines using the yeast expression system, 5xM2e DNA constructed with the GCN4-oligomer stabilizing domain fused to the 5xM2e protein and 6x histidine tag at the C-terminus was cloned into the pPICZ vector (Life Technology, Grand Island, NY, USA). A linear form of recombinant plasmid was transformed into *Pichia pastoris* (strain GS115, ATCC20864) by electroporation and inoculated to BMGY medium (1% yeast extract, 2% peptone, 1.34% YNB, 1% glycerol, 100 mM potassium phosphate, pH 6.0) then incubated at 30 °C for 48 h. The culture medium was changed with 0.5% methanol containing BMGY, known as BMMY medium and methanol was subsequently added. Histidine-tagged 5xM2e protein was purified by affinity chromatography using the nickel-nitrilotriacetic acid (Ni^2+^-NTA) column. Soluble 5xM2e protein vaccines were characterized using mouse anti-M2e monoclonal antibody (mAb) 14C2 (Abcam, Cambridge, MA, USA) by Western blot and enzyme-linked immunosorbent assay (ELISA) respectively. The size of the 5xM2e protein(s) was observed with multiple higher bands, which are bigger than the expected molecular weight (~17–18 kDa). It is speculated that oligomers emerged due to the GCN4-oligomer stabilizing domain fused to the 5xM2e protein (Appendix Figure A1b). Influenza A/Philippines/2/82 (H3N2) and A/Vietnam/1203/2004 viruses were grown in embryonated chicken eggs and purified from hen egg embryonic fluid. Inactivated virus was produced by treatment with formalin (1:4000, *v*/*v*) as previously described in [26].

### 2.2. Immunization and Challenges of Mice

Female BALB/c mice aged 6 to 8 weeks (*n* = 10, Charles River Laboratories, Inc., Wilmington, MA, USA) were intramuscularly (i.m.) prime immunized with 25 μg of 5xM2e protein (Pro) or 10 μg of 5xM2e VLP (VLP) and then boost immunized with 25 μg 5xM2e protein (Pro) or 10 μg of 5xM2e VLP at weeks (weeks) 0 and 4 respectively. Blood samples were collected at 3 weeks after being twice immunized. Immunized mice were anesthetized by isoflurane (Baxter, Deerfield, IL, USA) and intranasally (i.n.) infected with 4 × 50% mouse lethal dose (4 × LD_50_) of A/Philippines/2/82 (H3N2) in 50 μL of PBS per mouse 11 weeks after boost immunization. Mice were daily monitored for 14 days to record body weight changes and survival rates or else sacrificed at 6 days post-infection (dpi). All animal studies were approved and conducted according to the guidelines of Georgia State University Institutional Animal Care and Use Committee (IACUC). This study was covered by IACUC protocols A14025, approved on 20 October 2014, and A18001, approved on 18 September 2017.

### 2.3. Lung Viral Titers

Lung viral titers were determined using fertilized chicken eggs, which were incubated for 10 days at 37 °C. Lung homogenates were serially diluted in PBS and inoculated into the allantoic sacs. Inoculated embryonated eggs were further incubated for 3 days and chilled in a cold room overnight. The allantoic fluids were harvested and transferred into 96-well V-bottom plates containing chicken red blood cells. The plates were left at room temperature for 30 min to determine viral titers preventing the red blood cell precipitation.

### 2.4. Sample Preparation and Antibody Responses

Bronchoalveolar lavage fluids (BALF), lung extracts, and mediastinal lymph node (MLN) were collected day 6 post infection. BALF were obtained by infusing 1 mL of PBS into the lung via the trachea using a 25-gauge catheter (Exelint International Co., Los Angeles, CA, USA). MLN cells were cultured in plates pre-coated with M2e peptides for 1 or 5 days at 37 °C. Antibody titers in immune sera, BALF, and lung specific for influenza virus were determined by enzyme-linked immunosorbent assay (ELISA) using human M2e peptide and inactivated influenza A/Philippines/2/82 virus (H3N2, iPhil) as coating antigens. Briefly, M2e peptide or iPhil (4 μg/mL) antigens were coated onto 96-well microtiter plates (Nunc, Rochester, NY, USA), followed by incubation overnight at 4 °C. The plates were washed with PBS containing 0.05% Tween 20 (PBST) and blocked with 1% bovine serum albumin in PBST. Samples were added to the M2e-coated plates and incubated for 1.5 h (immune sera, BALF, lung homogenates), 1 or 5 days (MLN cells). Horseradish peroxidase-conjugated goat anti-mouse IgG, IgG1, and IgG2a (Southern Biotech, Birmingham, AL, USA) were used as secondary antibodies, and then tetramethylbenzidine (TMB) (Sigma-Aldrich, St. Louis, MO, USA) was used as a substrate. The optical density at 450 nm (OD_450_) was measured using an optical spectrophotometer reader. The total antibody concentration was determined with respect to the quantitative standard curve by using purified IgG, IgG1, IgG2a antibodies.

### 2.5. Bone Marrow-Derived Dendritic Cell (BMDC) Culture and Cytokine ELISA

Bone marrow (BM) cells were harvested from the femur and tibia of BALB/c and red blood cells (RBC) removed using RBC lysis buffer (Sigma Aldrich, St. Louis, MO, USA). BM cells were cultured in completed RPMI 1640 with 20 ng/mL mouse recombinant GM-CSF (Invitrogen, Waltham, MA 02451, USA) for 7 days at 37 °C in a 5% CO_2_. The in vitro-enriched BMDC culture preparations obtain immature CD11c+ DC populations at 80–89% high purity. BMDCs were stimulated with 5xM2e protein or VLP and M1 VLP (0.3, 1, 5 μg/mL) for 24 h. The levels of cytokines were determined in BALF and in culture supernatants from BMDCs by ELISA. Briefly, interleukin-6 (IL-6), IL-10, tumor necrosis factor alpha (TNF-α), and interferon gamma (IFN-γ) kits (eBioscience, San Diego, CA, USA) were used to determine the level of cytokines in the BALF and culture supernatants according to the manufacturer’s procedures.

### 2.6. Enzyme-Linked Immunospot (ELISPOT) Assay

To determine the cytokine spot-forming cells, lung cells (2 × 10^5^/well) and splenocytes (5 × 10^5^/well) were added into multi-screen 96-well plates coated with anti-mouse IFN-γ capture monoclonal antibody (BD Biosciences, San Diego, CA, USA) in the presence of M2e peptide (SLLTEVETPIRNEWGSRSN, 5 μg/mL) for 48 h. Cytokine spot-forming cells were detected with biotinylated anti-mouse IFN-γ and developed alkaline phosphates-labeled streptavidin (BD Pharmingen, San Diego, CA, USA). The spots were visualized with 3,3-Diaminobenzidine (DAB, Invitrogen) substrates and counted using an ELISpot reader (BioSys, Miami, FL, USA).

### 2.7. Flow Cytometry

BAL cells were collected from the BALF. The lung tissues without perfusion were homogenized, passed through a strainer and then lung extracts were spun on 44/67% Percoll gradients at 2800 rpm, for 15 min. The layer containing cells between 44% and 67% Percoll gradients were collected for the analysis of the lung cells. MLN single cell suspensions were prepared using the frosted glass microscope slides for flow cytometry analysis, cell phenotypic markers were stained at 4 °C using monoclonal antibodies specific for CD3, CD4, CD11b, CD11c, CD19, CD45, F4/80, Ly6c, IgD, CD138, GL7, MHCII (eBioscience or BD Pharmingen, San Diego, CA, USA). For intracellular cytokine staining, BAL and lung cells were stimulated with synthetic human influenza A virus M2e peptides (SLLTEVETPIRNEWGSRSN) (5 μg/mL) for 5 h at 37 °C in the presence of Brefeldin A (BFA) (20 μg/mL). After stimulation, lymphocytes were stained with monoclonal antibodies for CD4 (CD4-PE/Cy5, BD Biosciences) and CD8 (CD8α-PE, Biolegend, San Diego, CA 92121, USA). BD Cytofix/Cytoperm Plus Kit was used to fix and permeabilize lymphocytes labelled with specific phenotypic marker antibodies. Intracellular staining of the permeabilized lymphocytes took place with IFN-γ cytokine antibody (anti-mouse IFN-r-APC/Cy7, BD Biosciences). All samples were analyzed on a LSR-II/Fortessa flow cytometer (BD Biosciences, San Diego, CA, USA) and analyzed using the Flowjo software (FlowJo V10, Tree Star, Inc., Ashland, OR 97520, USA)).

### 2.8. Protective Assay of Immune Sera

Naïve or immune sera were incubated at 56 °C for 30 min and mixed with a 10× LD50 of reassortants A/Vietnam/1203/2004 H5N1 (rgH5N1) virus. Naïve mice were inoculated with the mixture of sera (25 μL of 2-fold diluted sera) and virus. Mice were daily monitored for 14 days to record body weight changes and survival rates.

### 2.9. Intraperitoneal Injection to Determine Acute Immune Responses

BALB/c mice were intraperitoneally (i.p.) injected with 200 μL of PBS, 5xM2e VLP (10 μg), and 5xM2e protein (25 μg). Sera and peritoneal exudates were collected at 2 or 24 h post-injection (p.i.) for ELISA assay. The infiltrating cells at the injection site were determined in the peritoneal cells by flow cytometry.

### 2.10. Statistics

All results are presented as the mean ± the standard errors of the mean (SEM). The statistical significance for all experiments was performed by one or two-way analysis of variance (ANOVA). Prism software (GraphPad Software, Inc., San Diego, CA, USA) was used for all data analysis. The comparison used to generate a P value is indicated by horizontal lines (*; *p* < 0.05, **; *p* < 0.01, ***; *p* < 0.001).

## 3. Results

### 3.1. The 5xM2e VLP but Not the 5xM2e Protein Is Immunogenic and Effective in Eliciting IgG2a Isotype Antibodies

**Figure 1:**
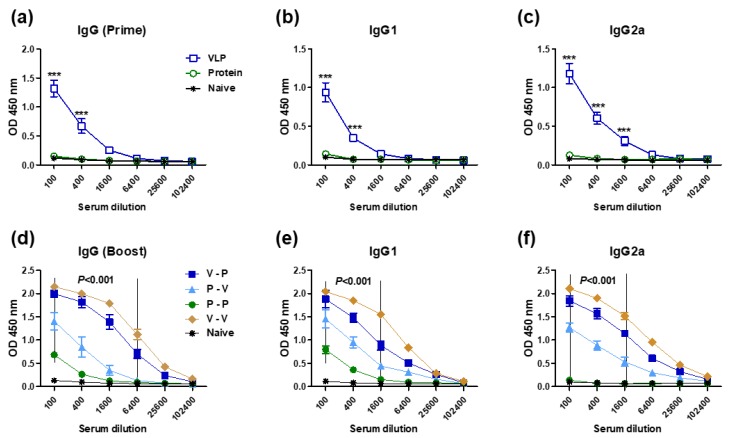
VLP is superior to protein in priming M2e specific IgG and IgG2a isotype antibodies. BALB/c mice (*n* = 5 per group) were intramuscularly immunized with VLP (10 μg) or protein (25 μg) to 5xM2e at weeks 0 and 4. M2e-specific antibody responses were determined in prime or boost immune sera collected at 3 weeks after immunization by ELISA. IgG, IgG1, and IgG2a in prime (**a**–**c**) and boost (**d**–**f**) immune sera. VLP: 5xM2e VLP prime, Protein: 5xM2e protein prime, V-P: VLP prime/protein boost, P-V: protein prime/VLP boost, P-P: protein prime boost, V-V: VLP prime boost. The statistical significance was determined by a two-way ANOVA and a Bonferroni posttest. Error bars indicate the means ± SEM of concentration from individual animals. ***; *p* < 0.001 indicates statistical significance between VLP prime group (VLP or V - P) and protein prime group (P-P or P – V).

**Figure A2:**
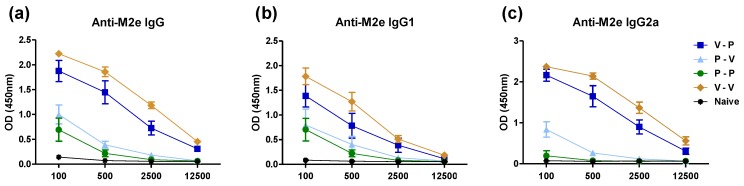
Anti-M2e antibody responses in immune sera collected before challenges. M2e-specific antibody responses were determined in boost immune sera collected at 10 weeks after immunization by ELISA. IgG (**a**), IgG (**b**), and IgG2a (**c**) in immune sera. V: 5xM2e VLP, P: 5xM2e protein, V-P: VLP prime/protein boost, P-V: protein prime/VLP boost, P-P: protein prime boost, V-V: VLP prime boost.

The impact of VLP versus protein platforms presenting M2e epitopes on priming immune responses is not yet fully understood. This study focused on carrying out comparative studies on 5xM2e VLP and 5xM2e protein vaccines. The 5xM2e proteins purified from yeast cells displayed 64-fold higher reactivity to 14C2 Mab specific for M2e than the 5xM2e VLP vaccines produced in insect cells (Appendix Figure A1). To determine the immunogenic properties of 5xM2e vaccines, BALB/c mice (*n* = 10 per group) were immunized with 5xM2e VLP (10 μg) or 5xM2e protein (25 μg). Mice primed with 5xM2e VLP (VLP) induced M2e-specific IgG, IgG1, and IgG2a antibodies at significantly higher levels than those in mice primed with 5xM2e protein, which did not induce M2e antibodies at detectable levels (Figure 1a–c). To compare the prime effects in terms of boosting the immune responses, a strategy of heterologous prime boosting was applied. The 5xM2e protein homologous prime-boost (P-P) induced low levels of IgG and IgG1, and a detection limit level of IgG2a isotype antibodies (Figure 1d–f). Heterologous protein prime and VLP boost (P-V) resulted in substantial levels of IgG, IgG1, and IgG2a isotype antibodies, which were similar to those in mice with 5xM2e VLP prime only (VLP). A reverse order of heterologous VLP prime and protein boost (V-P) developed significantly higher levels of IgG antibodies, 64-fold (IgG), 16-fold (IgG1), and over 25,000-fold (IgG2a) higher compared to the homologous P-P group (Figure 1d–f). The IgG2a isotype antibody levels in the V-P group were comparable to those in the homologous VLP prime boost (V-V) group whereas the IgG1 antibody was slightly higher by V-V immunization than that by V-P immunization (Figure 1e,f, Appendix Figure A2). The highest levels of IgG antibodies were induced in the V-V group (Figure 1d–f, Appendix Figure A2). In our previous study of V-V vaccination, IgG antibodies recognizing human, swine, and avian type I M2e antigens were similarly induced in mice immunized with 5xM2e VLP [23]. These results suggest that 5xM2e VLP is highly effective in priming IgG2a isotype antibody responses that can be boosted by low immunogenic 5xM2e protein.

### 3.2. Heterologous VLP Prime and Protein Boost Improves Protection Compared to Protein-Only Immunizations

**Figure 2:**
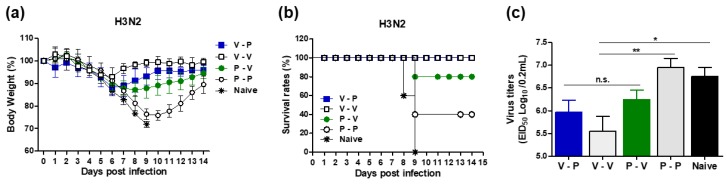
The 5xM2e VLP primed mice display enhanced protection against H3N2 virus. Immunized mice (*n* = 10 per group) were infected with a lethal dose of A/Philippine/2/1982 (H3N2) at 11 weeks after boost immunization. Lung viral titers were detected by inoculating chicken eggs. EID_50_, 50% egg infections dose. (**a**) Body weight changes. (**b**) Survival rates. (**c**) Lung viral titers. Naïve: naïve mice infected with influenza virus, V-P: VLP prime/protein boost, P-V: protein prime/VLP boost, P-P: protein prime boost, V-V: VLP prime boost. The statistical significance was confirmed by one-way ANOVA and Dunnett’s multiple comparison test. Error bars indicate the means ± SEM of concentration from individual animals. *; *p* < 0.05, **; *p* < 0.01. n.s.; no significance.

**Figure A3:**
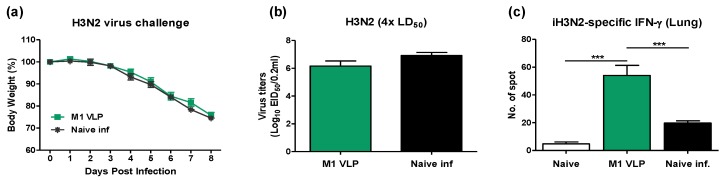
Protective effets by M1 VLP immunization. V1 VLP immunized mice (n = 8 per group) were infected with a lethal dose of A/Philippines/2/1982(H3N2) at 6 weeks after boost immunization. (**a**) Body weight changes. (**b**) Lung viral titers were detected by inoculating chicken eggs. EID_50_ 50% egg infections dose. (**c**) The spot number of IFN-γ secreting cells were determined by ELIASpot. The statistical significance was confirmed by one-way ANOVA and Dunnett’s multiple comparison text. Error bars indicate the means ± SEM of pooled samples from each group of animals.***: *p* < 0.001.

To determine the protective efficacy after heterologous prime boost immunization with 5xM2e VLP or 5xM2e proteins, mice were challenged with a lethal dose of H3N2 virus (A/Philippines/82, 4× LD_50_) at 11 weeks post-boost. Naïve mice showed a severe body weight loss of >25% and all mice died of infection by nine days post infection (dpi) (Figure 2a,b). Protein prime-boost mice (P-P) exhibited 20% to 25% weight loss resulting in 40% survival rates and surviving mice gradually recovered their weight. In contrast, heterologous VLP and protein (V-P) immune mice displayed significantly less weight loss around 10% and all (100%) survived the lethal challenge (Figure 2a,b). Notably, protein prime VLP boost (P-V) immune mice revealed 13% weight loss, slower recovery, and 80% survival rates. VLP prime-boost mice (V-V) showed 8% weight loss and 100% protection (Figure 2c). To determine the efficacy of controlling lung viral loads, viral titers were measured in lung lysates at day six post-challenge by an egg infectious titration assay. Naïve or protein prime-boost (P-P) mice showed high levels of viral loads (7.5 × 10^7^ EID_50_). Heterologous VLP or protein immunizations resulted in 100-fold lower viral loads (7.5 × 10^5^ EID_50_ for V-P mice) and 10-fold lower (6.3 × 10^6^ EID_50_) for P-V mice. The VLP prime-boost (V-V) group showed the lowest lung vital titers (5.5 × 10^5^ EID_50_) (Figure 2d). The mice that were immunized with M1 VLP did not show any significant protection against A/Philippines H3N2 virus as evidenced by there being no significant difference in weight change compared to unvaccinated naïve mice after infection (Appendix Figure A3). The lung viral titers were not reduced in M1 VLP immunized mice compared to those in naïve mice that were infected with H3N2 virus. These results suggest that 5xM2e VLP (but not M1 VLP) primed mice are effective in conferring protection and controlling lung viral loads.

### 3.3. The 5xM2e Immune Sera from VLP-Primed but Not Protein-Primed Mice Confer Protection

**Figure 3:**
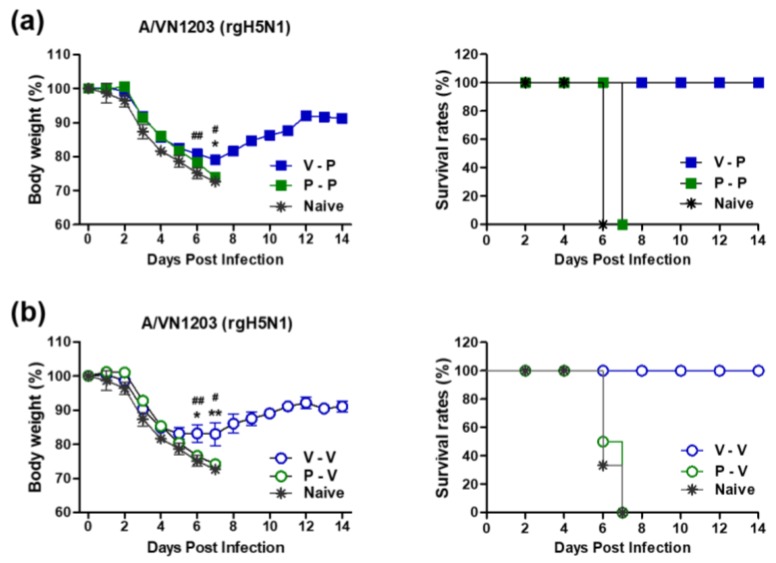
5xM2e VLP-primed but not protein-primed immune sera confer cross-protection against rgH5N1 virus. Mice (*n* = 3) were intranasally infected with a high lethal dose (10× LD_50_) of reassortant rgH5N1 (A/VN1203 (rgH5N1)) virus mixed with each immune serum collected at three weeks after boost immunization. Body weight changes were monitored for 14 days. (**a**,**b**) Body weight changes and survival rates. Naïve: naïve mice infected with influenza virus, V-P: VLP prime/protein boost, P-V: protein prime/VLP boost, P-P: protein prime boost, V-V: VLP prime boost. The statistical significance was confirmed by two-way ANOVA. Error bars indicate the means ± SEM of concentration from individual animals. *; *p* < 0.05, **; *p* < 0.01 in comparison to V-P and P-P, ^#^; *p* < 0.05, ^##^; *p* < 0.01 in comparison to V-P and naïve.

Since M2e-specific antibodies do not neutralize viruses, we applied a modified passive immunization and in vivo protection assay of sera in naïve mice as reported in our previous studies. Naïve mice were infected with a mixture of immune sera and a lethal dose of rgH5N1 virus and monitored for morbidity and mortality. Naïve mice that received a mixture of rgH5N1 virus and naïve sera or immune sera from 5xM2e protein prime boost (P-P) and 5xM2e protein prime VLP boost (P-V) mice were not protected, as evidenced by severe body weight loss and the fact that all died of infection at 7 dpi (Figure 3a,b). In contrast, naïve mice with immune sera from VLP-primed (V-P, V-V) mice showed 17 to 21% weight loss and gradually recovered from a lethal dose of rgH5N1 virus, resulting in 100% survival rates (Figure 3a,b). These data suggest that 5xM2e VLP-primed but not 5xM2e protein-primed immune sera confer protection against lethal rgH5N1 influenza virus infection.

### 3.4. The 5xM2e VLP Is Superior to 5xM2e Protein in Inducing B Cell Responses

**Figure 4:**
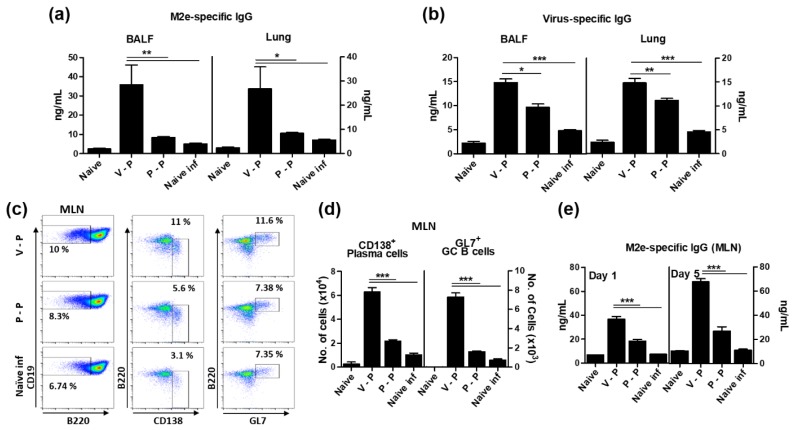
VLP priming is superior to protein in inducing mucosal IgG antibodies, plasma, and germinal center phenotypic B cells. Bronchoalveolar lavage fluids (BALF), lung lysates (Lung), and mediastinal lymph node (MLN) cells were collected at 6 dpi (A/Phil, H3N2). Antigen-specific IgG levels were determined in BALF and lung lysates by ELISA. (**a**,**b**) IgG specific to M2e peptide or virus antigens in the BALF and lung. (**c**) Representative flow cytometry profiles. The cells were gated from pre-gated mature B cells (CD19^+^ IgD^−^ B220^+/−^). (**d**) CD138^+^ plasma and GL7^+^ germinal center (GC) B cells in the MLN. The cells were stained with anti-CD138 and -GL7 antibodies. (**e**) M2e-specific IgG. MLN cells were cultured in the plates coated with M2e peptide for one or five days. The groups are the same as in Figure 3. The statistical significance was confirmed by one-way ANOVA and Dunnett’s multiple comparison test. Error bars indicate the means ± SEM of concentrations from individual animals. *; *p* < 0.05, **; *p* < 0.01, ***; *p* < 0.001 indicate statistical significance between the groups as marked by the bars.

To determine whether 5xM2e VLP or 5xM2e protein prime-boost would induce local humoral immune responses correlating with enhanced protection, antigen-specific antibody levels were determined in the bronchoalveolar lavage fluids (BALF) and lung lysates of immunized mice at 6 dpi (Figure 4a,b). Consistent with serum antibody responses, IgG antibodies specific for M2e and inactivated virus (iPhil, H3N2) were induced at significantly higher levels in the BALF and lungs of VLP-primed mice (V-P) than those of protein-primed (P-P) or naïve infection mice (Figure 4a,b). Furthermore, B cell activation and the generation of CD138^+^ plasma cells and the germinal center (GC) phenotypic GL7^+^ B cells (CD19^+^ B220^+^) were analyzed by flow cytometry in the mediastinal lymph nodes (MLN) of immunized mice at 6 dpi. THe CD138^+^ plasma cells and GL7^+^ GC B cells in VLP-primed mice (V-P) were 3-fold and 4.5-fold higher than protein-primed mice (P-P), respectively (Figure 4c,d). We also determined the in vitro antibody production as a measure of the VLP priming effects on generating antigen-specific, antibody-secreting cell responses. MLN cells were collected at 6 dpi and cultured in 96-well plates coated with M2e peptides. Significant amounts of M2e specific IgG antibodies were produced in MLN from VLP-primed mice (V-P) compared to those of protein-primed (P-P) or naïve infected mice at 1 or 5 days after in vitro culture, while 2- to 2.5-fold lower levels of IgG antibodies were observed in P-P mice (Figure 4e). Therefore, these results provide evidence that 5xM2e VLP is superior to 5xM2e protein in priming B cell responses in mucosal sites of viral replication and draining lymph nodes.

### 3.5. VLP-Primed Mice Prevent Excessive Dendritic Cell Recruitment upon Virus Infection

**Figure 5:**
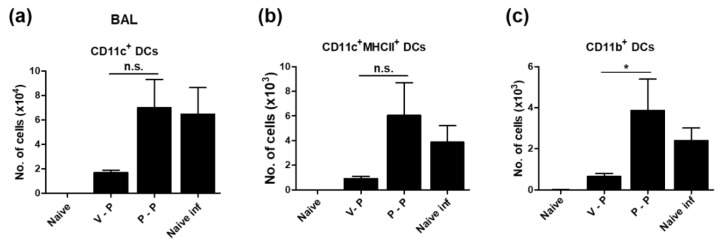
VLP-primed mice show lower levels of infiltrating dendritic cells after virus challenge. The cells obtained from BALF at 6 dpi (A/Phil, H3N2) were stained with anti-CD11c, CD11b, MHCII, CD45 to determine dendritic cell subtypes by flow cytometry. Each DC subtype was gated from the pre-gated CD45^+^ F4/80^-^ cells. (**a**,**b**) Total CD11c^+^ DCs and activated DCs (CD11c^+^ MHCII^+^). (**c**) CD11b^+^ DCs gated from CD11c^+^ MHCII^+^ DCs. The group labels are the same as in the Figure 3. The statistical significance was confirmed by one-way ANOVA and Dunnett’s multiple comparison test. Error bars indicate the means ± SEM of concentrations from individual animals. *; *p* < 0.05. n.s.; no significance.

We next analyzed the cellularity of dendritic cells (DCs) in the lung at 6 dpi by flow cytometry and observed a notable difference between VLP- and protein-primed mice. VLP-primed mice (V-P) showed significantly lower levels of conventional CD11c^+^ DCs, activated CD11c^+^ MHCII^+^ DCs, and CD11b^+^ DCs, with four-fold to six-fold lower levels compared to protein-primed (P-P) or naïve infected mice (Figure 5a–c), suggesting a protective correlation with lower lung viral titers in the VLP-primed group.

### 3.6. The 5xM2e VLP Platform Is More Effective in Inducing Effector CD4 T Cell Immune Responses than Protein

**Figure 6:**
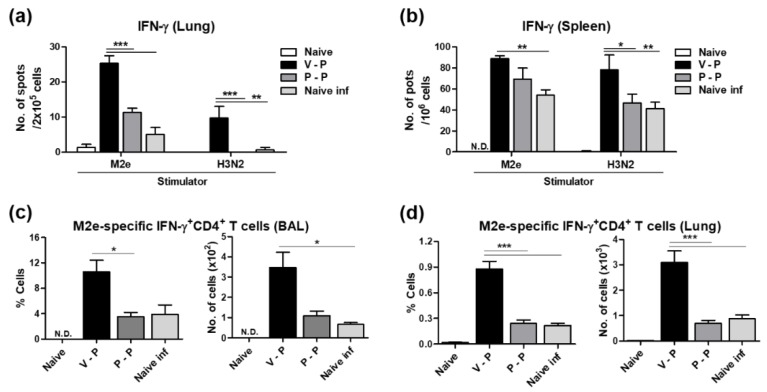
VLP prime is superior to protein prime in inducing antigen-specific T cells producing IFN-γ. The cells were harvested from the spleen and lungs of immunized mice 6 dpi (A/Phil, H3N2). (**a**,**b**) The spots producing IFN-γ were determined by culture for 2 days in the presence of M2e peptide or H3N2 virus antigen using ELISpot analysis. The data were obtained from averages of pooled samples with triplicates of each group. (**c**,**d**) The CD4 T cells producing IFN-γ were determined after culture for 5 h in the presence of M2e peptide. Antigen-specific T cells were detected by intracellular cytokine staining with anti-IFN-γ, anti-CD4 antibody. The group labels are the same as in the Figure 3. The statistical significance was confirmed by two-way ANOVA and a Bonferroni post-test or one-way ANOVA and Dunnett’s multiple comparison test. Error bars indicate the means ± SEM of cellularity from individual animals. *; *p* < 0.05, **; *p* < 0.01, ***; *p* < 0.001.

We determined whether VLP priming in V-P mice would be effective in inducing M2e-specific effector T cell responses. Lung cells and splenocytes harvested at 6 dpi were stimulated with M2e peptides or inactivated H3N2 virus (A/Phil). The results of an IFN-γ ELISpot assay indicate that VLP-priming in V-P mice induced significantly higher numbers of IFN-γ secreting cells specific to M2e or virus in the lungs and spleens than those from protein-priming in P-P mice (Figure 6a,b).

To better understand the importance of 5xM2e VLP priming effects on T cell responses, the cells from BALF and lungs at 6 dpi were subjected to intracellular cytokine staining and flow cytometry analysis after stimulation with M2e peptides. The VLP-primed group (V-P) showed significantly higher numbers of IFN-γ^+^ CD4^+^ T cells in the BAL and lung samples than those of the protein-primed (P-P) group (Figure 6c,d).

### 3.7. The 5xM2e VLP Is More Effective in Stimulating DCs In Vitro to Secrete Inflammatory Cytokines than 5xM2e Proteins

**Figure 7:**
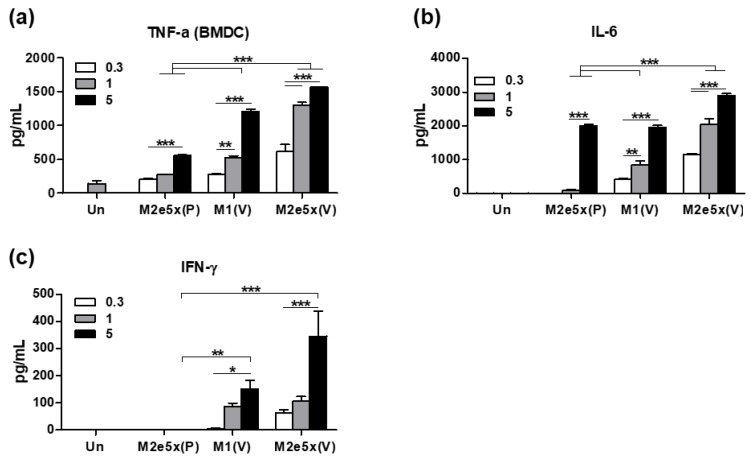
VLP effectively stimulates BMDCs in vitro to secrete cytokines. BMDCs were stimulated with M2e5x, M1 VLP, or protein (0.3, 1, 5 μg/mL) for 24 h. (**a**–**c**) The levels of cytokines (**a**) TNF-α, (**b**) IL-6, (**c**) IFN-γ were determined in the culture supernatants by ELISA assay. Un: Medium only, P: proteins, V: VLP. The statistical significance was confirmed by one-way ANOVA and Dunnett’s multiple comparison test. Error bars indicate the means ± SEM of concentrations from individual animals. *; *p* < 0.05, **; *p* < 0.01, ***; *p* < 0.001 indicate statistical significance between the groups as marked by the bars.

It is possible that 5xM2e VLP might have an intrinsic property of stimulating DCs to secrete T helper type 1 (Th1) cytokines. BMDCs were in vitro incubated with 5xM2e VLP or 5xM2e proteins at different concentrations and cytokine levels in culture supernatants were determined by cytokine ELISA kits (Figure 7). Significantly higher levels of cytokines including TNF-α, IL-6, and IFN-γ were produced from 5xM2e VLP-stimulated or M1 VLP-stimulated BMDC cultures in a dose-dependent manner than those from 5xM2e protein-stimulated BMDCs (Figure 7a–c). TNF-α cytokine was detected in 5xM2e protein-stimulated BMDCs at 3–5-fold lower levels than VLP-cultured BMDCs (Figure 7a). A high concentration (5 μg/mL) of 5xM2e protein was needed to stimulate BMDCs to secrete IL-6, whereas a low concentration of 5xM2e VLP could stimulate BMDCs to secrete cytokines. IFN-γ was detected at high levels in BMDC cultures after 5xM2e VLP or M1 VLP stimulation, but not with 5xM2e protein stimulation (Figure 7b,c). Therefore, these results provide support for the idea that 5xM2e VLP or M1 VLP is more effective in activating DCs to secrete Th1 cytokines than 5xM2e proteins, in correlation with the induction of Th1 type IgG2a antibodies and CD4 T cell responses in 5xM2e VLP-primed mice.

### 3.8. VLP Is Effective in the Acute Induction of Cytokines and Recruiting Innate Immune Cells

**Figure 8:**
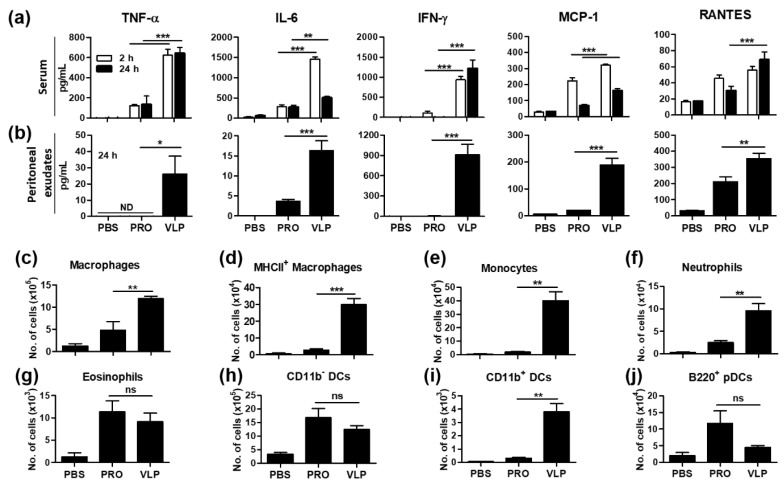
VLP is superior to protein in priming acute innate immune responses in vivo at the injection site. Balb/c mice (*n* = 3) were i.p. injected with 200 μL of PBS, 10 μg 5xM2e VLP, or 25 μg 5xM2e protein. The levels of cytokine and chemokine were determined in sera and peritoneal exudates collected at the indicated times. The levels of cytokines (TNF-α, IL-6, IFN-γ) and chemokines (MCP-1, RANTES) in serum (**a**) and Peritoneal exudates (**b**). (**c**–**j**) Different phenotypic innate immune cells were determined in the cells collected from peritoneal exudates at 24 h post injection using flow cytometry. PRO: 5xM2e proteins, VLP: 5xM2e VLP. The statistical significance was confirmed by one-way ANOVA and Dunnett’s multiple comparison test. Error bars indicate the means ± SEM of concentrations from individual animals. *; *p* < 0.05 **; *p* < 0.01, ***; *p* < 0.001 indicate statistical significance between the 5xM2e protein and VLP groups as marked by the bars. n.s.; no significance.

Innate immunity plays a key role in the induction and regulation of adaptive immune responses. To better understand the effects of vaccines in inducing acute innate immunity at the site of injection, mice were intraperitoneally (i.p.) injected with 5xM2e VLP or protein. Sera were obtained at 2 and 24 h after the injection, then the mice were sacrificed to collect peritoneal exudates. Cytokines and chemokines in sera and peritoneal exudates were determined by ELISA (Figure 8a,b). VLP injection of mice induced significantly higher levels of proinflammatory cytokines (TNF-α, IL-6, IFN-γ) and chemokines (MCP-1, RANTES) in the sera (Figure 8a) and peritoneal exudates (Figure 8b) than those by measured after the 5xM2e protein injection of mice. Furthermore, we determined innate immune cell types and cellularity in the peritoneal cavity at 24 h post injection of mice with either VLP or protein vaccine (Figure 8c–j). Consistent with inducing higher inflammatory intermediates, VLP-injected mice showed significant higher infiltration of innate immune cells such as macrophages, monocytes, neutrophils, and CD11b^+^ DCs into the peritoneal cavity (Figure 8c–f,i) whereas the 5xM2e protein group showed the recruitment of eosinophils, CD11b^-^ DCs, and B220^+^ plasmacytoid DCs (pDCs) at high levels (Figure 8g,h,j). These results suggest that 5xM2e VLP is effective in generating locally inflammatory microenvironment and in recruiting innate immune cells, which would contribute to inducing stronger adaptive immunity than protein vaccine.

## 4. Discussion

Influenza M2e antigens have poor immunogenic properties as shown by there being no detectable IgG antibodies after the tandem repeat 5xM2e protein prime immunization at a high dose (25 ug) in the absence of adjuvants. This study investigated whether the presentation of 5xM2e on a VLP platform (5xM2e VLP) would overcome the poor immunogenic properties of M2e and the prime immune responses that could be boosted by 5xM2e protein vaccination. The 5xM2e VLP was found to be superior to 5xM2e protein in developing humoral and cellular immune responses, as well as in conferring protection. The 5xM2e VLP but not the protein primed-immune responses were effectively boosted by subsequent 5xM2e protein vaccination. This study is consistent with a clinical finding that individuals with detectable pre-existing M2 antibodies are likely to see boosted M2e immune responses after a follow-up influenza infection [2]. The results in this study provide insight for the design of vaccine constructs and vaccination strategies.

Licensed human hepatitis B virus and papillomavirus vaccines are based on the concept of VLP, although they are non-enveloped. Avian and pandemic influenza-enveloped VLP vaccines produced in insect cells were shown to be safe and efficacious in clinical trials [27,28,29]. The frequency and levels of M2e antibodies are low in humans particularly in populations of a young age [2,3]. Antibodies specific to M2e were not observed at substantial levels after influenza vaccination [30] or live virus infection [2,3,23,31]. In this aspect of difficulty in inducing M2e specific antibodies, it is significant to effectively induce M2e antibodies by 5xM2e VLP priming, which can be boosted by poor immunogenic antigens such as M2e proteins or influenza vaccination.

The 5xM2e VLP is an assembled structure with membrane envelopes presenting 5xM2e in an anchor form on the surface at higher levels than M2 proteins in influenza virus [23]. We reported the comparative efficacy experiments and outcomes in a previous study, comparing inactivated split HA-based vaccination and 5xM2e VLP vaccination after homologous virus and heterosubtypic virus challenge [32]. Inactivated virus-based vaccination conferred better protection against HA-matching homologous virus than 5xM2e VLP vaccination [32]. However, 5xM2e VLP vaccine was superior to inactivated virus vaccination in conferring cross-protection and a future pandemic virus challenge, when vaccinated mice were challenged with HA-mismatching heterosubtypic virus [32]. This study highlights the unique immunogenic properties of the 5xM2e VLP vaccine platform in comparison with 5xM2e proteins. The reactivity of 5xM2e proteins to M2e mAb (14C2) was significantly higher, over 64-fold time higher than 5xM2e VLP (Appendix Figure A1). Thus, the 5xM2e protein vaccine appears to have higher M2e epitope levels per μg proteins than 5xM2e VLP. Despite higher levels of M2e epitopes, the 5xM2e protein was so poor in immunogenicity and unable to effectively induce M2e antibodies after the prime vaccination of mice even with a high 25 μg dose. M2e protein conjugate vaccines required multiple vaccinations at higher vaccine doses in the presence of potent adjuvants to induce M2e antibodies [13,33]. The display of repetitively organized antigenic molecules on the surfaces of VLPs was attributed to the effective stimulation of the immune system [34,35].

In this study, 5xM2e VLP was able to prime immune responses that were effectively boosted by subsequent poor immunogenic 5xM2e protein vaccination (VLP/protein versus protein/protein). The 5xM2e VLP was highly effective in inducing both IgG1 and IgG2a isotype antibodies, but prime-boost with 5xM2e protein induced only IgG1 at low levels. Interestingly, 5xM2e VLP-primed mice effectively boosted IgG2a (Th1 type) antibodies in response to 5xM2e protein vaccination. The 5xM2e VLP (but not protein) priming might have generated a pool of primed memory B cell and T cell Th1 responses and their activation thresholds might be low enough to be boosted after exposure to Th2-biased protein vaccination. The reverse order of 5xM2e protein priming and 5xM2e VLP boost resulted in IgG antibodies similar to those by 5xM2e VLP priming only, suggesting the minimum and limited effects of the 5xM2e protein in priming immune responses. The type of immune response, such as non-neutralizing M2e immunity, might have an impact on the protective efficacy. IgG2a antibodies are known to interact efficiently with Fc receptors by virtue of their Fc domain properties [36,37]. Also, Fc receptors were demonstrated to play an important role in conferring protection mediated by M2e immunity [23,38]. Therefore, the induction of IgG2a antibodies by 5xM2e VLP is considered to contribute to effective viral clearance, probably via activation of the Fc-receptor-mediated complementary system or the stimulation of antibody-dependent cellular cytotoxicity [39]. Consistent with these previous studies, the group of 5xM2e VLP prime and 5xM2e protein boost was more effective in lowering lung viral loads, inducing Th1 type cellular immune responses, and in conferring protection than the 5xM2e protein prime boost vaccination.

H5 HA VLP, which contains hemagglutinin proteins from A/Vietnam/03/2004 (H5N1) was shown to be more immunogenic and protective than the five-fold higher doses of recombinant H5 HA protein vaccines in mice [40]. It was also reported that VLP vaccine containing pandemic 2009 H1 HA conferred better protection in ferrets than commercial split vaccine [41]. The repetitive and organized antigens on VLPs are effective in activating B cell receptors, leading to enhanced humoral immune responses and triggering Toll-like receptors (TLRs), driving a class switch to the IgG2a isotypes [39,42]. MyD88, an innate TLR signaling adaptor molecule was shown to play a critical role in inducing the IgG2a isotype, IFN-γ secreting Th1 immune responses, long-lived plasma cells, and in conferring protection after VLP vaccination [43]. We observed a severe in vivo defect in inducing Th1 type immune responses and conferring protection in HA VLP-vaccinated MyD88 KO mice [43].

Natural influenza virus infection induces strong humoral and cellular immunity in surviving hosts. High levels of viral replication in the lung produces strong inflammatory signals resulting in recruiting inflammatory innate immune cells and antigen presenting cells. Migratory DC populations are recruited into the lung in response to the inflammatory signals due to influenza virus infection. Nonetheless, it is still the subject of intense ongoing research which pulmonary DC subset is responsible for antigen presentation to T cells and inducing immunity against influenza virus infection. Both CD11b^+^ and CD103^+^CD11b^−^ migratory DC subsets were shown to be responsible for presenting antigens to naive CD8 T and CD4 T cells [44,45,46]. Plasmacytoid B220+ DC subsets were shown to be implicated in promoting anti-influenza B cell immunity [47]. We found that conventional CD11c^+^ DCs, activated CD11c^+^ MHCII^+^ DCs, and CD11b^+^ DCs were highly recruited into the lung in the P-P group or unvaccinated naïve mice after influenza virus infection. High lung viral replication in these less-protected mice is assumed to be responsible for recruiting diverse DC subset populations, whereas the protected mice with better control of lung viral replication prevented the infiltration of DC subsets, probably due to low inflammatory signals. Further studies are required regarding which pulmonary DC subset is responsible for antigen presentation to naïve or memory T cells after influenza A virus infection.

In this study, 5xM2e VLP-primed mice showed higher levels of plasma cells and GC B cells in addition to M2e specific IgG antibodies and IFN-γ secreting CD4 T cells in mucosal sites at an early time (6 dpi) post-challenge. The stimulation of antigen-presenting cells is critical for priming adaptive cellular responses. Particulate antigens such as VLPs are relatively effective in stimulating antigen-presenting cells leading to the priming of T cells [48]. Our in vitro studies found that 5xM2e VLP is more effective in stimulating BMDCs to secrete inflammatory cytokines (TNF-α, IL-6, IFN-γ) than 5xM2e protein. Notably, 5xM2e VLP is more effective in enhancing the production of acute inflammatory cytokines (TNF-α, IL-6, IFN-γ) and chemokines (MCP-1, RANTES), as well as the infiltration of macrophages, monocytes, neutrophils, and CD11b^+^ DCs at the site of injection by in vivo stimulation. Cytokines and chemokines are important mediators of immunoregulation in the site of vaccination and infection. TNF-α is a master proinflammatory cytokine produced by macrophages that augments the expression of other pro-inflammatory cytokines and chemokines [49]. IL-6 has been shown to modulate inflammation and affect adaptive immunity—IL-6-deficient mice are more susceptible to influenza infection [50]—and to promote humoral responses by activating Th cells [51]. MCP-1/CCL2 and RANTES/CCL5 are known to promote the recruitment of monocytes and immature DCs [52] and to stimulate T cell proliferation; mice lacking RANTES exhibit hampered T cell proliferation and IFN-γ and IL-2 secretion following antigen stimulation [52]. Taken together, the unique structural and immunogenic properties of VLP platform vaccines likely contribute to priming superior immune responses and conferring protection compared to soluble protein antigens.

## 5. Conclusions

This study presents data supporting superior immunogenic properties of 5xM2e VLP over 5xM2e proteins although 5xM2e proteins contain high density of antibody reactive antigenic epitopes compared to 5xM2e VLP. There was no correlation between in vitro antigenic property and *in vivo* capability of antigens to develop immune responses. Priming with immunogenic 5xM2e VLP vaccines would provide immunologic benefits to boost immune responses to poor immunogenic 5xM2e protein vaccination later. 

## Figures and Tables

**Table 1:** The 5xM2e sequence and challenge virus.

5xM2e Vaccine	M2e Sequences (2–20)	Challenge Virus	M2
Human (2×)	SLLTEVETPIRNEWGSRSN (consensus)	H3N2 (A/Philippines)	Human M2
Swine (1×)	SLLTEVETPTRSEWESRSS (A/California/4/2009)	rgH5N1 (A/PR8 backbone)	Human M2
Avian I (1×)	SLLTEVETPTRNEWESRSS (most common)		
Avian II (1×)	SLLTEVETLTRNGWGCRCS (second common H5, H9)		

Note: The M2e sequences of human type, swine type, and avian type I have changes of cysteine to serine (CRCS to SRSS) to avoid undesirable disulfide bonds.
